# The Zhongshan Score: A Novel and Simple Anatomic Classification System to Predict Perioperative Outcomes of Nephron-Sparing Surgery: Erratum

**DOI:** 10.1097/01.md.0000464828.75442.31

**Published:** 2015-04-10

In the article “The Zhongshan Score: A Novel and Simple Anatomic Classification System to Predict Perioperative Outcomes of Nephron-Sparing Surgery”,^1^ which appeared in Volume 94, Issue 5 of *Medicine*, Dr. Wang's email address was spelled incorrectly. The correct email address is wang.hang@zs-hospital.sh.cn. The article has since been corrected online.
